# Adjustment of refugee children and adolescents in Australia: outcomes from wave three of the Building a New Life in Australia study

**DOI:** 10.1186/s12916-018-1124-5

**Published:** 2018-09-04

**Authors:** Winnie Lau, Derrick Silove, Ben Edwards, David Forbes, Richard Bryant, Alexander McFarlane, Dusan Hadzi-Pavlovic, Zachary Steel, Angela Nickerson, Miranda Van Hooff, Kim Felmingham, Sean Cowlishaw, Nathan Alkemade, Dzenana Kartal, Meaghan O’Donnell

**Affiliations:** 1 Phoenix Australia, Melbourne, Victoria Australia; 2 Department of Psychiatry, University of Melbourne, Melbourne, Victoria Australia; 3 Liverpool Hospital, Sydney, NSW Australia; 4 School of Psychiatry, University of New South Wales, Sydney, NSW Australia; 5 ANU Centre for Social Research and Methods, Australian National University, Canberra, Australian Capital Territory Australia; 6 School of Psychology, University of New South Wales, Sydney, NSW Australia; 7 Centre for Traumatic Stress Studies, University of Adelaide, Adelaide, SA Australia; 8 Black Dog Institute, Sydney, NSW Australia; 9 St John of God Hospital Richmond, Sydney, Australia; 10 Melbourne School of Psychological Sciences, University of Melbourne, Melbourne, Victoria Australia; 11 Population Health Sciences, Bristol Medical School, University of Bristol, Bristol, UK; 12 Monash Health, Melbourne, Victoria Australia

**Keywords:** Mental health, Adjustment, Strengths and Difficulties Questionnaire, Psychosocial, Ecological, Refugee, Children, Adolescents, Resettlement

## Abstract

**Background:**

High-income countries like Australia play a vital role in resettling refugees from around the world, half of whom are children and adolescents. Informed by an ecological framework, this study examined the post-migration adjustment of refugee children and adolescents 2–3 years after arrival to Australia. We aimed to estimate the overall rate of adjustment among young refugees and explore associations with adjustment and factors across individual, family, school, and community domains, using a large and broadly representative sample.

**Methods:**

Data were drawn from Wave 3 of the Building a New Life in Australia (BNLA) study, a nationally representative, longitudinal study of settlement among humanitarian migrants in Australia. Caregivers of refugee children aged 5–17 (*N* = 694 children and adolescents) were interviewed about their children’s physical health and activity, school absenteeism and achievement, family structure and parenting style, and community and neighbourhood environment. Parent and child forms of the Strengths and Difficulties Questionnaire (SDQ) were completed by caregivers and older children to assess social and emotional adjustment.

**Results:**

Sound adjustment according to the SDQ was observed regularly among young refugees, with 76-94% (across gender and age) falling within normative ranges. Comparison with community data for young people showed that young refugees had comparable or higher adjustment levels than generally seen in the community. However, young refugees as a group did report greater peer difficulties. Bivariate and multivariate linear regression analyses showed that better reported physical health and school achievement were associated with higher adjustment. Furthermore, higher school absenteeism and endorsement of a hostile parenting style were associated with lower adjustment.

**Conclusions:**

This is the first study to report on child psychosocial outcomes from the large, representative longitudinal BNLA study. Our findings indicate sound adjustment for the majority of young refugees resettled in Australia. Further research should examine the nature of associations between variables identified in this study. Overall, treating mental health problems early remains a priority in resettlement. Initiatives to enhance parental capability, physical health, school achievement and participation could assist to improve settlement outcomes for young refugees.

## Background

As of end-2017, 68.5 million people globally were displaced due to war and political conflict, of whom 25.4 million were recognised as refugees according to the United Nations High Commissioner for Refugees (UNHCR) (http://www.unhcr.org/en-au/figures-at-a-glance.html). More than half of all displaced people are children and adolescents. High-income countries such as Australia play important roles in the long-term resettlement of refugees, both as individuals and families (http://www.unhcr.org/en-au/figures-at-a-glance.html).

As the pressure for high-income countries to resettle greater numbers of refugees and families increases, there is a growing imperative to understand and support the well-being and emotional health of individuals and families admitted – a prerequisite for positive settlement outcomes. To date, however, there is a dearth of high quality empirical research involving representative samples that investigates the psychosocial well-being of children and adolescents within resettled families in high-income countries, and factors in the post-settlement environment associated with sound adjustment (i.e. social and emotional functioning) [1].

Prior research into the mental health and psychosocial adjustment of young refugees post-migration has yielded varying results. Systematic reviews of epidemiological studies in high-income countries estimate the prevalence of post-traumatic stress disorder, anxiety and depression to be between 19% and 54%, 33% and 50%, and 3% and 30%, respectively, among young refugees [2]. Generally, these prevalence statistics are elevated in comparison with community norms [2, 3]. The differences in prevalence of disorders across studies have been attributed to methodological variations, including sample differences (e.g. clinical, community or convenient, older vs. younger), variations in measures and diagnostic assessments (e.g. self-report vs. clinical measures, cut-off points), sampling characteristics (e.g. length of time since conflict or in resettlement) [4], cultural variations in expressions of distress [5], and specific factors relating to subsamples of refugees (e.g. higher vs. lower torture experience) [6].

The mixed findings regarding prevalence present an unresolved paradox. On the one hand, there is at least tentative consensus that the majority of refugee youth experience low level or no mental health or adjustment difficulties [7, 8]. On the other hand, it may be expected that the traumas and adversities these individuals have experienced place them at heightened risk of traumatic stress problems [9]. What is lacking from the existing body of research is a robust estimate from representative samples of how many young refugees are well adjusted and how many are not, which children will experience adjustment problems, and what factors are associated with adjustment in young refugees. Characteristics of the post-settlement environment are likely to play a key role in influencing the adjustment outcomes of refugee children and adolescents. They may also help to explain the observed variation in prevalence of psychological difficulties.

Adopting an ecological framework can assist in identifying and assessing the multiple factors that are associated with adjustment among young refugees. Bronfenbrenner’s [10] original ecological framework considered child well-being within influential systems – the micro system (the day-to-day and inner relationships surrounding the child), the meso system (the network of relationships between micro systems, such as between parents and teachers), the exo system (the more remote social settings that have indirect effects on the child such as neighbourhood) and the macro system (the broader social, cultural and political beliefs, ideals, and customs that incorporate the micro, meso and exo systems) [11]. This conceptual framework has increased awareness of the risk and protective context of the child in terms of not only individual characteristics but also family, school, peer and community environments [10, 12]. In refugee populations, ecological models have been called for to improve the understanding of the health and wellbeing needs of these communities [11, 13].

The application of ecological models to young refugees suggests that a constellation of stressors from a range of domains contributes to mental health and adjustment following displacement, over and above the impact of prior war exposure [9, 13]. It is widely acknowledged that post-migration factors are important determinants of mental health outcomes in resettled adult refugee samples [14]. These can be as powerful as, or even more so, than pre-migration experiences of war-related trauma and loss in predicting mental health outcomes [15, 16]. Less is known about the significance of different post-migration environments for child and adolescent adjustment. A number of studies have suggested, however, that factors such as poor housing, insufficient financial support, language acquisition difficulties and racism, can all affect the mental health outcomes of this population [17–19].

Multiple domains have been shown to influence adjustment in young refugees, including those relating to the individual, family, school, peers and the wider community. Individual characteristics such as age, physical health and pre-migration trauma experiences are important personal and historical risk factors [16, 20]. Additionally, family factors including supportive, warm and nurturing parent-child relationships [21, 22], as well as a positive family life and unity [23], are thought to impact on the adjustment of young refugees. Among school and peer factors, support from friends and positive school experiences have been identified as indicators of adjustment among school-aged children [17], while community factors such as integration into the host society have also been associated with positive mental health outcomes among migrants and refugees [15, 16]. Consistent with this literature, one illustrative systematic review adopted an ecological model to highlight the prospective mental health risks associated with individual factors (e.g. female gender), family factors (e.g. parental mental health) and community factors (e.g. discrimination and racism) [20].

A major problem in past studies conducted with refugees relates to methodological issues associated with non-random and convenience sampling. This can result in either an under-estimation of distress (i.e. samples composed predominantly of healthy participants) or over-estimation (i.e. samples composed predominantly of individuals in need of support) [6], and limits what can be reasonably concluded and generalised about the refugee population [24]. Further evidence from representative samples is therefore required to help determine the psychosocial adjustment of refugee youth post-settlement, as well as the environmental factors that help explain or are related to these adjustment outcomes. This is particularly important given the potential for constructive screening and intervention during this crucial post-settlement period.

Post-settlement environments, including the policies and interventions in place to support refugee resettlement, vary enormously across countries. Australia for example, is highly regarded for the level of support provided to resettled humanitarian entrants (e.g. housing support, language acquisition and healthcare), but until now there has not been data available that speak to the adjustment outcomes of young refugees resettled in Australia. Gaining insight into the relative level of psychosocial adjustment in this population, and factors associated with better or poorer adjustment, is thus crucial to inform targeted policy and intervention strategies. This article is the first to report on levels of psychosocial adjustment and factors associated with optimal adjustment among a broadly representative sample of resettled child and adolescent refugees in Australia.

The aim of this study was to examine adjustment in a child and adolescent refugee cohort resettled in Australia 2 to 3 years post-migration. Specifically, we aimed to estimate the proportion of young refugees who are well/maladjusted, and to compare their adjustment with age and gender equivalent community norms. To further assist in understanding the factors associated with the observed adjustment of young refugees, a second aim was to explore the individual, familial, school, and community risk and protective factors associated with adjustment. This may then enable the identification of potential targets for intervention across these domains.

A key contribution of this study is the examination of a cohort that is broadly representative of the refugee population in Australia, allowing for a more robust examination of adjustment outcomes than has been previously possible. To enable this, we use data from the Building a New Life in Australia (BNLA) study [1]. In previous longitudinal studies that followed young refugees through to adulthood in resettled countries (the United States, Canada, Denmark, Sweden and Australia [25–30]), sample sizes were relatively small, selective or unrepresentative of contemporary youth refugee cultural groups. To our knowledge, the BNLA project is the first and largest longitudinal prospective cohort study of refugees and their families in Australia, and one of the largest in the world.

In light of the time-restricted context in which data collection in the BNLA study took place, and in the absence of available follow-up data on refugee children and adolescents at this stage (follow-up data collection is ongoing), we focus specifically on putative risk factors for early adjustment and those that are potentially modifiable (i.e. factors that fall within the remit of resettlement services in high-income countries) in the post-settlement period. The factors investigated included individual factors (age, gender, physical health and physical activity), familial factors (family structure and parenting approach), school factors (achievement and absenteeism), and community factors (extracurricular engagement, perceived support within the community, perception of safety and friendliness of the resident neighbourhood). We use the term adjustment in this study to refer to the general social and emotional functioning of young refugees.

## Methods

### The BNLA study and data source

The child/adolescent sample investigated in this study is derived from the BNLA study, undertaken by the Australian Government Department of Social Services and the Australian Institute of Family Studies [31]. The main BNLA study is described below, while the child and adolescent sample recruited at Wave 3 is described thereafter.

The BNLA is a population-based cohort study tracing the settlement outcomes of individuals and families over five waves, commencing from the point of being granted a permanent humanitarian visa [32]. Recruitment and Wave 1 occurred between October 2013 and February 2014, while subsequent waves of data have been collected annually. To date, four waves of data have been collected, with data from the first three waves released so far. The present data pertains to Wave 3, undertaken between October 2015 and February 2016, which was the first wave that collected information relating to children and adolescents.

### BNLA sampling and participants

BNLA participants were recruited from 11 sites in Australia covering major cities and regional areas. These sites were selected to ensure an adequate sample size to allow for robust analyses, based on the concentration of eligible refugees in particular localities, appropriate geographic spread and an optimal representation of holders of different types of humanitarian visas granted in Australia. Participants in the BNLA study comprised ‘principal’ and ‘secondary’ applicants for a humanitarian visa in Australia that was granted in the period preceding the study. Principal applicants were the main applicants within a migrating unit (typically a family), whereas secondary applicants were other members of the migrating unit (e.g. child, spouse, other adult family member). Initial eligibility criteria included (1) being a ‘principal applicant’ for a humanitarian visa that was granted 3 to 6 months prior to the survey (i.e. May to December, 2013) and already holding a permanent protection visa (the ‘offshore’ group), or granted a permanent protection visa in the previous 3 to 6 months after arrival in Australia by boat or on another visa type such as a tourist visa (the ‘onshore’ group); and (2) being 18 years or older. Seventy-eight percent of migrating units had followed an ‘offshore’ pathway while the remaining 22% followed an ‘onshore’ pathway. During the initial recruitment phase, principal applicants provided consent for other members of their migrating unit to be contacted. These ‘secondary applicants’ were invited to participate if they were (1) at least 15 years of age and (2) residing with the principal applicant. Although the gender of principal and secondary applicants varied, in most cases the secondary applicant was female.

To contextualise Australia’s humanitarian intake programme, those who arrive via offshore pathways typically include UNHCR identified and referred refugees, global humanitarian special programme refugees (i.e. living outside Australia and home countries but subject to gross human rights violations, nominated by a person or organisation in Australia), in-country special humanitarian cases, emergency rescue and women at risk cases, and immediate family members of people already granted protection in Australia. For those who arrive via onshore pathways (i.e. arrivals by boat or via other means such as student/tourist visas), there may be a period of waiting for an application for a humanitarian visa to be assessed. As Australia’s laws require the detention of non-citizens who are in Australia without a valid visa, those who arrive via onshore pathways may spend time in community detention or immigration detention. Refugee camp experiences may vary across these humanitarian visa classes.

### BNLA data collection procedures

During Wave 1 and 3, BNLA data were collected at home visits. In alternate waves, data were collected via telephone. Surveys were administered by field workers using a computer-assisted self-interview, which enabled participants to respond privately to self-report questions using a computer interface. Participants could opt instead to complete a computer-assisted personal interview, whereby field interviewers asked questions displayed on a screen and entered responses. Computer-assisted self-interviews lasted 45 minutes on average, while computer-assisted personal interviews took just over 60 minutes on average, to complete. Survey materials were available in nine languages following translation and multi-stage quality assurance review. In most cases, participants were matched with an interviewer who was a native speaker of their preferred language. Where this was not possible or desired, participants could opt to use an accredited interpreter.

### BNLA Wave 3: child and adolescent sampling

Wave 3 data was collected between October 2015 and February 2016 and included interviews with 1155 principal applicants and 739 secondary applicants. For 87% of the sample, this time point corresponded to a residency period of 2 to 3 years in Australia. Nine percent of participants had spent 3 or more years living in Australia, and 4% had spent between 1 and 2 years in Australia.

Wave 3 was the first time in the BNLA study that a child module was included as a nested component of the broader study. This module targeted children and adolescents in the migrating unit aged 5 to 17 years. It incorporated two components. The first was a primary caregiver report, which was completed by participants (principal or secondary visa applicants) who identified as the primary caregiver in the migrating unit. The second component was a child self-report, which was completed by older children and adolescents (aged 11 and 17 years).

Recruitment of the child and adolescent sample purposively targeted older children (11–17 years) over younger children (5–10 years) to maximise the number of child participants able to provide self-report data. Up to two children per household were invited to participate. Initial sampling occurred by randomly selecting two children between 11 and 17 years of age in each migrating unit. In households with multiple children, but only one child between 11 and 17 years, the eldest child was recruited as well as one randomly selected younger child between 5 and 10 years. In households with exclusively younger children, two children between 5 and 10 years were randomly selected. There were no unaccompanied children in the sample. Caregivers were invited to complete the caregiver report with respect to the children selected for recruitment. Only children recruited to the study between 11 and 17 years of age were invited to complete the child self-report module, which was administered via pencil and paper.

**Fig. 1:**
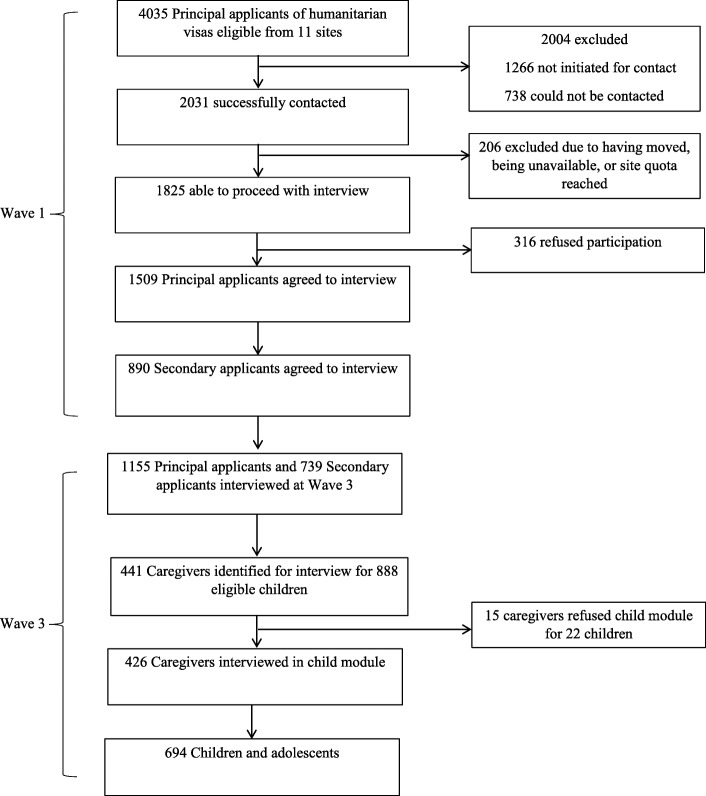
Flow of participants through the BNLA study from Wave 1 to Wave 3

Of the 888 eligible children, data were collected for 694 children and from 426 primary caregivers, of whom *n* = 310 were mothers (72.8%), *n* = 97 were fathers (22.8%), and *n* = 19 (4.4%) were other members of the migrating unit/household (primarily siblings). Figure 1 summarises the recruitment process, outlining the flow of participation by adults (principal and secondary applicants) recruited in Wave 1 and subsequent recruitment of caregivers and children and adolescents in Wave 3.

### Measures

#### The child module

The child module was developed by the BNLA study team in consultation with members of the current author group, who are experts in refugee mental health and longitudinal research. The development process prioritised psychosocial factors significant to refugee settlement that could be assessed within the time available for data collection. The caregiver report component of the module was administered to caregivers of children aged 5–17 years and assessed perceptions of the child’s overall physical health and activity, school participation (absenteeism) and achievement, language use, mental health and emotional symptoms, and adjustment. It also incorporated a structured parenting questionnaire. The child self-report was administered to children aged 11–17 years and included a questionnaire assessing physical health and activity, engagement in extracurricular activities, and self-reported adjustment. The child module required 10 minutes per child to complete.

### Social and emotional adjustment

#### Strengths and Difficulties Questionnaire (SDQ) – Parent and child form

The SDQ [33] was used to assess refugee child and adolescent adjustment. The SDQ comprises 25-items that operationalise five subscales, namely emotional symptoms, conduct problems, hyperactivity/inattention, peer problems and prosocial behaviour [33]. There are parent and child report versions available, which ask how true each item is for the nominated child (or in the case of the child version, for him/herself) over the past 6 months. Items are scored on a 3-point Likert scale (0 = not true, 1 = somewhat true, or 2 = certainly true). With the exception of prosocial behaviour, item scores were aggregated to generate a total difficulties score (range 0–40), with higher scores indicating increased adjustment problems [34].

The SDQ is not a diagnostic measure, yet it can discriminate between children from high- and low-risk samples and screen for child psychiatric disorders, including in non-Western populations [34, 35]. The SDQ is available in more than 20 languages and is one of the most widely used dimensional assessment instruments in multicultural research [36]. It has demonstrated acceptable to strong internal consistency [37, 38] and adequate test-retest reliability [38] with refugee samples in humanitarian settings and has been used widely with child and adolescent refugees in high-income countries [30, 39–47]. Evidence for the reliability of the SDQ with refugee samples is available from Canada, where the measure demonstrated satisfactory to high internal consistency [48]. In the present study, caregivers completed the SDQ–parent form for children aged 5–17 years and children aged 11–17 completed the SDQ–child form.

For children aged 5–10 years, we analysed the SDQ caregiver report data, given that the parent/caregiver report is the most reliable index of adjustment for a younger age group [49]. For children aged 11–17, the SDQ self-report data were analysed given the increased validity of self-report data in this age group. In analysing SDQ data, children aged 5–17 were assigned to categories for ‘normal’, ‘borderline’ or ‘abnormal’ on subscales and total difficulties, based on the online English language cut-off scores [50]. We also compared SDQ scores of refugee children and adolescents in this sample with Australian norms. These norms (means and standard deviations), broken down by age groups, are outlined in the results section. Specifically, age groupings in this study enabled comparison to Australian norms, across three groups as follows: (1) 5–10 years old, (2) 11–13 years old and (3) 14–17 years old.

### Domain measures

**Fig. 2:**
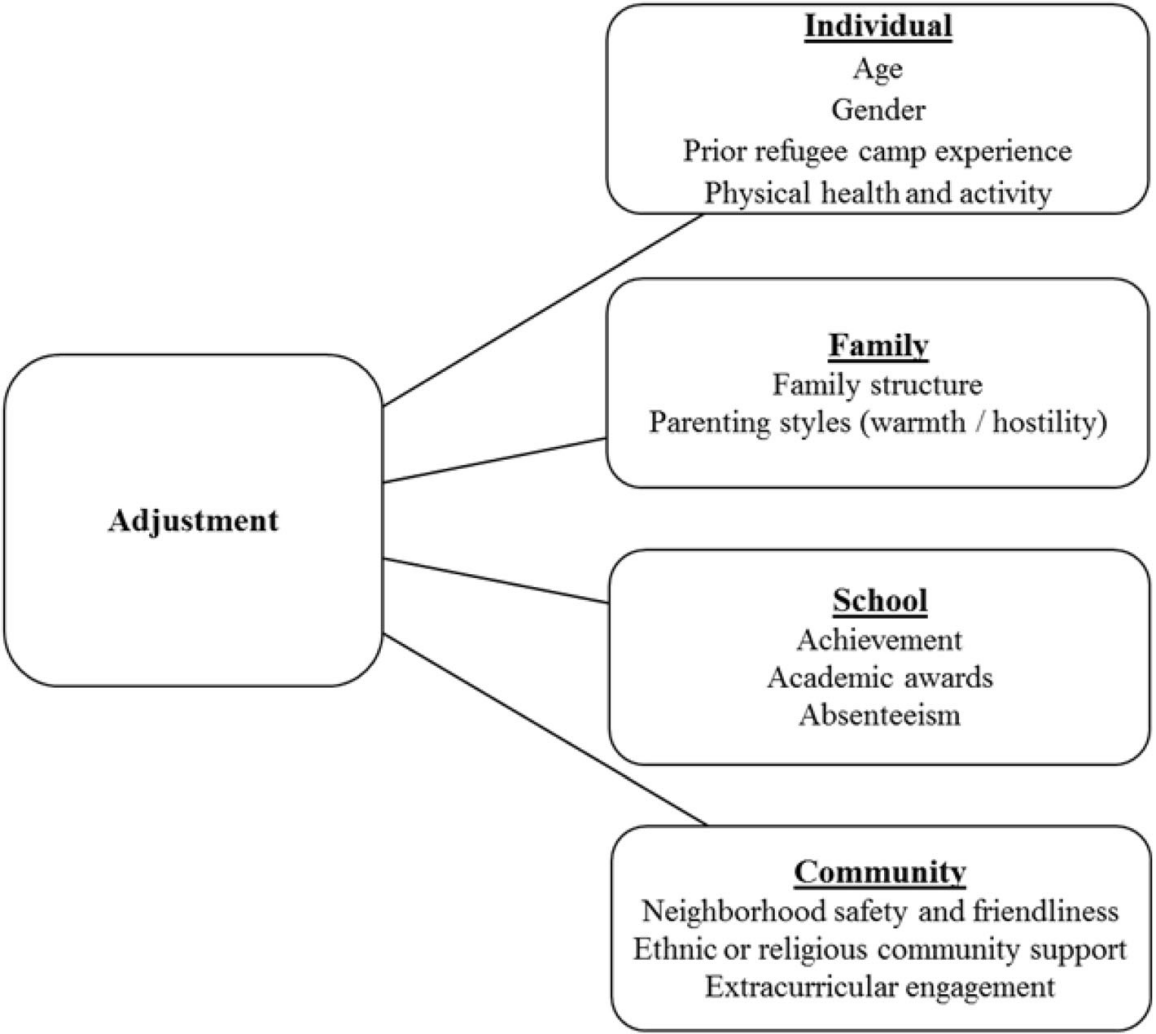
Domains and corresponding variables of interest in the current study in relation to young refugees’ adjustment

A summary of domains and variables examined in relation to refugee youth adjustment is presented in Fig. 2. Caregivers of refugee children and adolescents completed the following indices (except where noted as having been completed via young person self-report). Measures were also based on survey administration at Wave 3, except where specified. The origin of these questions (with exception to questions specific to refugee experience) are based on the Growing Up in Australia: The Longitudinal Study of Australian Children study. This is a major longitudinal study following the development of 10,000 children and families from all areas of Australia, which includes items with origins in validated health and developmental screening measures [51].

#### Individual domain

##### Background and pre-migration experiences

Sociodemographic measures were administered at Wave 1 and included items about child age and gender. During this wave, caregivers were also asked: “*Did you spend any time in a refugee camp before you came to Australia?*” If they answered yes, they were also asked: “*How long did you spend there?*”

##### Parent-rated child health and physical activity

Parent-rated child health was measured using caregiver reports to a single-item measure: “*In general, would you say* [named child]'s *health is (1) excellent, (2) very good, (3) good, (4) fair, or (5) poor*”? Caregivers were also asked about their child’s physical activity: “*In the last 7 days, how many days has* [named child] *done a total of 60 minutes or more of physical activity, which was enough to raise their breathing rate?*” The latter was scored using an open response format.

#### Family domain

##### Family structure

An indicator of family structure was defined on the basis of information reported by the principal applicant, which identified the relationship of all household members to themselves (e.g. spouse, unrelated child, grandchild, biological child). This allowed for a classification of family structure in terms of whether the principal applicant was in a couple or single, and whether other family members lived in the household.

##### Parenting warmth and hostility

Parenting warmth and hostility were measured using caregiver responses to 10 questions [52]. Examples of warmth questions included: “*How often do you have warm close times together with this child?*” and “*How often do you enjoy listening to this child and doing things with him/her?*” Examples of hostility questions included: “*I have been angry with this child*” and “*I have raised my voice*”. Responses were based on a 5-point Likert scale, with 1 = Never/Almost Never, 5 = Always/Almost always.

#### School domain

##### School achievement and absenteeism

Caregivers with children who were enrolled in school were asked: “*How would you describe* [named child]'s *overall achievement at school?*” Responses were based on a 5-point Likert scale, with scores dichotomised such that 0 = Excellent/Above average/Average achievement, and 1 = Below average/Well below average achievement. School absenteeism was measured by caregiver responses to: “*During the previous four weeks of school how many days has* [named child] *been absent?*” This item was scored using an open response format.

##### School award

Children aged 11–17 were also asked: “*In the last year, have you won any awards or been recognised for doing well in certain activities?*” Response options included (1) won an academic award, (2) received a community service award, (3) been selected to represent the school, (4) received an award in sports, or (5) received an award in music, arts, dance performance or drama.

#### Community domain

##### Extracurricular activities/engagement

Children aged 11 and over were asked about participation in extracurricular activities: “*In the last 6 months, have you regularly attended any of these activities?*” Responses included (1) individual sport, (2) team sport, (3) musical instruments or singing, (4) ballet or other dance, or (5) religious group. Respondents were required to circle as many activities as were applicable.

##### Ethnic/religious/community support

Community support was measured using caregiver responses to the following question: “*Do you feel that you have been given support/comfort in Australia from* (a) *your national or ethnic community; and* (b) *your religious community?*” Responses were measured on a 3-point scale (Yes/Sometimes/No).

##### Neighbourhood friendliness and safety

Caregivers were asked to provide responses to statements about their neighbourhood (local area), including (1) “*The people in my neighbourhood are friendly*” and (2) “*I feel safe in my neighbourhood*”. Responses were scored on a 4-point Likert scale, with 1 = Strongly agree and 4 = Strongly disagree.

### Data analysis

Data-file preparation was conducted in SPSS Version 25 and included management of data regarding children aged 5–10 years, which were obtained from caregiver reports, and regarding those aged 11–17 years, which were obtained from both caregivers and child self-report. This process was guided by the following principles (except where otherwise specified): (1) if responses from both caregiver and child reports were available (as was the case for children aged between 11 and 17 years), then self-reported data from children were used (e.g. on SDQ and SDQ subscales); (2) where data was available from child self-reports only (e.g. regarding extracurricular engagement) then this information was analysed; and (3) where information was available from caregiver reports only (e.g. about family structure) then this information was analysed.

In the first stage of analyses, descriptive statistics were produced to summarise the sociodemographic profile of the sample, as well as the distribution of measures across individual, family, school and community domains. Total and subscale scores on the SDQ were produced and reported separately for boys and girls, across three age groups (5–10, 11–13, and 14–17 years). This enabled comparison with age-equivalent Australian norms (http://www.sdqinfo.com/norms/AusNorm.html). Comparisons were based on examinations of SDQ mean scores and categorised indicators (‘normal’, ‘borderline’ or ‘abnormal’), defined using the online English language cut-off scores [50], through use of independent group *t* tests and χ^2^ tests, respectively. These were conducted in SPSS and incorporated Wave 3 cross-sectional survey weights to adjust for initial non-response and subsequent attrition, and to ensure that estimates reflected the population characteristics of refugees receiving humanitarian visas. Comparisons with age/gender equivalent community norms were analysed using SPSS modules that allowed for clustering within families (the intraclass correlation coefficient for the SDQ was 0.59).

The second stage of analyses comprised a series of regression models which examined the post-migration variables associated with children’s adjustment difficulties. These models were estimated using MPlus Version 7.4, using robust maximum likelihood and multiple imputation across *k* = 100 datasets to manage item-level missing data. As such, these analyses did not incorporate information about survey weights, but did account for clustering within families using the TYPE = COMPLEX function. A series of bivariate models were estimated initially, which specified the SDQ total score as the endogenous variable, and considered exogenous independent variables across individual, family, school and community domains. Regression parameter estimates were standardised by the variance of exogenous and endogenous variables and were reported along with 95% confidence intervals. All independent variables were also entered into a single multiple regression model to examine and minimise the risk of confounded associations.

## Results

### Demographics

An exact breakdown of sample characteristics, including gender distributions, can be found in Table 1, for both the overall sample and by age groups. There was a fair representation of children across age groups and genders (53% male, 47% female). Most participants reported Iraq or Afghanistan as their country of origin (38.8% and 23.7%, respectively), followed by Bhutan (11.1%), Myanmar (8.1%), Iran (6.4%), or ‘Other’ (11.9%). Common primary languages spoken were Arabic (21.7%), Assyrian Neo-Aramaic (20.9%), Persian (13.4%), and Nepali (11.4%), followed by Dari (9.2%), Hazaraghi (9.0%), Burmese and related (6.2%), or Chaldean Neo-Aramaic (3.1%). The remaining 5.3% of the sample reported other languages. The majority of children and adolescents reported using both English and their caregivers’ language, although they were more likely to use the caregivers’ language at home (χ^2^(1, *N* = 669) = 48.86, *p* < 0.001). The majority of the sample (96%) had spent less than 12 months in Australia prior to being recruited to the study, representing a relatively early settlement group.

**Table 1:** Means, standard deviations and frequencies for demographic and domain (individual, family, school, community) variables investigated in this study of young refugees (*N* = 694)

	Total group	Age category		
		5–11 years	11–13 years	14–17 years
Demographics^a^				
N	597 (52.9% male)	216 (52.3% male)	169 (53.2% male)	212 (53.0% male)
Age (M, SD)	11.6 (3.6)	7.5 (1.7)	12.0 (0.81)	15.5 (1.1)
*Often use carer language*	69.7%	66.8%	70.7%	71.8%
*Often use English*	82.3%	84.8%	82.9%	79.2%
Individual domain				
*Physical health*^*a*^				
Rating of physical health (range 1–5)	4.05 (0.04)	4.08 (0.07)	4.12 (0.07)	3.96 (0.07)
Physical activity in past week (days)	2.49 (0.10)	2.61 (0.18)	2.67 (0.17)	2.27 (0.14)
Family domain				
*Family structure*^*a*^				
Couple with children under 18	45.0%	73.2%	54.0%	19.3%
Couple with children under 18 and other family	32.6%	18.3%	23.2%	47.9%
Single with children under 18	9.1%	6.7%	8.4%	11.3%
Single with children under 18 and other family	13.3%	1.7%	14.5%	21.5%
*Parenting style*^*a*^				
Parenting warmth (range 5–25)	20.42 (3.77)	21.27 (3.31)	20.30 (4.03)	19.62 (3.83)
Parenting harshness (range 5–25)	9.22 (3.72)	8.76 (3.08)	9.72 (4.27)	9.29 (3.81)
School domain				
School achievement average or above average^a^	93.9%	94.0%	94.0%	93.6%
Achievement award^b^	22.7%	–	21.2%	23.8%
Absenteeism (days per 4 weeks)^a^	1.13 (0.11)	0.87 (0.14)	0.94 (0.13)	1.45 (0.21)
Community domain				
Extracurricular engagement^b^	87.5%	–	88.2%	86.9%
*Neighbourhood feels safe*^*a*^				
Agree	96.5%	98.3%	94.7%	95.9%
Disagree	3.5%	1.7%	5.3%	4.1%
*Neighbourhood friendly*^*a*^				
Agree	96.7%	98.7%	93.4%	96.9%
Disagree	3.3%	1.3%	6.6%	3.1%
*Ethnic or religious community support*^*a*^				
Yes	32.6%	34.8%	34.5%	30.0%
Sometimes	21.9%	24.3%	19.9%	21.1%
No	45.5%	41.0%	45.6%	49.0%

Note. Values are reported in the form of either M(SD) or percentages where indicated. Based on weighted data

^a^Caregiver-reported information

^b^Child-reported information

### Descriptive findings across individual, family, school and community domains

Table 1 shows ratings provided by caregivers and older children and adolescents (age 11–17) on factors measured across domains. This information is summarised below.

#### Individual domain findings

As depicted in Table 1, most children had ‘very good’ physical health ratings (mean 4.05) and engaged in at least 1 hour of intense physical activity on average 2.5 days a week (mean 2.49). In both the 11–13 and 14–17 age groups, boys reported significantly more physical activity than girls.

#### Family domain findings

More than three-quarters of refugee children and adolescents were from dual caregiver households, with a high proportion also living with other family members. Due to the complexity of how responses to the relevant BNLA questions were itemised, it was not possible to state whether caregivers from dual households were both biological parents; however, it is reasonable to infer that this is likely. The composition of families was similar across Wave 1 and Wave 3, whereby 72.2% of families were in couple caregiver households and 25.9% in single caregiver households in Wave 1, compared to 71% and 28.5%, respectively, in Wave 3. In relation to caregiver parenting style, caregivers reported relatively high scores on warmth and lower scores on hostility.

#### School domain findings

The vast majority of caregivers reported that their children were at or above average for school achievement. Among older students, around 22.7% self-reported being the recipient of an achievement award from their school. Over half the sample (54.6%) reported no school absenteeism in the last 4 weeks, with 19.6% reporting only 1 day of absenteeism and 8.3% reporting at least 1 day per week absenteeism on average.

#### Community domain findings

Most children aged 11 years or older reported extracurricular activities. There was a gender difference in the reporting of extracurricular activities in the 14–17 age group, where a lower proportion of girls (80.9%) reported participating in extracurricular activities compared with boys (92.2%, χ^2^(1, *N* = 231) = 6.07, *p* = 0.01). At Wave 3, most caregivers described their wider local Australian community as safe (96.5%) and friendly (96.7%). In response to questions regarding ethnic or religious community support, 32.6% of caregivers rated their ethnic or religious community as ‘supportive’, 21.9% as ‘sometimes supportive’, and 45.5% as ‘not supportive’.

### Adjustment outcomes

#### Overall adjustment

Table 2 shows findings regarding this study’s main outcome of interest – social and emotional adjustment, as measured by SDQ total scores and for the five SDQ subscales. Findings are presented according to age groups (5–10, 11–13 and 14–17 years of age), as compared with mean scores of Australian age-matched norms.

**Table 2:** Strengths and Difficulties Questionnaire (SDQ) mean total and subscale scores for young refugees and comparison with Australian norms

	Boys			Girls		
	Parent SDQ			Parent SDQ		
	*BNLA (5–10)*	*AUS (7 to 10)*		*BNLA (5–10)*	*AUS (7 to 10)*	
*Aged 5–10*	*n* = 109	*n* = 160	*t* test *p*	*n* = 97	*n* = 197	*t* test *p*
Total difficulties	10.1 (0.59)	9.9 (0.51)	0.733	8.7 (0.56)	7.7 (0.41)	0.074
Emotional symptoms	2.4 (0.22)	2.3 (0.17)	0.794	2.0 (0.23)	2.3 (0.14)	0.264
Conduct problems	1.7 (0.16)	1.8 (0.13)	0.439	1.3 (0.17)	1.3 (0.11)	0.908
Hyperactivity/inattention	3.6 (0.23)	4.1 (0.21)	0.037*	3.1 (0.21)	2.6 (0.16)	0.023*
Peer problems	2.4 (0.17)	1.8 (0.16)	0.000***	2.3 (0.16)	1.5 (0.14)	0.000***
Prosocial behaviour	7.7 (0.23)	8.0 (0.14)	0.146	8.3 (0.22)	8.7 (0.11)	0.097
	Self SDQ			Self SDQ		
	*BNLA (11–13)*	*AUS (11–13)*		*BNLA (11–13)*	*AUS (11–13)*	
*Aged 11 to 13*	*n* = 86	*n* = 148	*t* test *p*	*n* = 81	*n* = 144	*t* test *p*
Total difficulties	8.6 (0.57)	8.8 (0.45)	0.779	8.8 (0.65)	8.0 (0.51)	0.198
Emotional symptoms	2.3 (0.21)	2.0 (0.16)	0.147	3.0 (0.26)	2.6 (0.18)	0.155
Conduct problems	1.4 (0.15)	2.0 (0.15)	0.000***	1.4 (0.22)	1.3 (0.12)	0.754
Hyperactivity/inattention	2.6 (0.21)	3.2 (0.19)	0.008*	2.3 (0.20)	2.6 (0.18)	0.179
Peer problems	2.4 (0.20)	1.7 (0.13)	0.001***	2.2 (0.19)	1.4 (0.13)	0.000***
Prosocial behaviour	8.1 (0.18)	7.8 (0.16)	0.115	8.3 (0.17)	8.6 (0.12)	0.058
	*BNLA (14–17)*	*AUS (14–17)*		*BNLA (14–17)*	*AUS (14–17)*	
*Aged 14 to 17*	*n* = 127	*n* = 115	*t* test *p*	*n* = 112	*n* = 144	*t* test *p*
Total difficulties	8.1 (0.41)	10.1 (0.56)	0.000***	10.3 (0.57)	9.1 (0.40)	0.036*
Emotional symptoms	2.1 (0.17)	2.1 (0.19)	0.788	3.2 (0.24)	2.9 (0.16)	0.152
Conduct problems	1.3 (0.13)	2.4 (0.18)	0.000***	1.8 (0.14)	1.7 (0.12)	0.559
Hyperactivity/inattention	2.6 (0.14)	4.0 (0.22)	0.000***	2.6 (0.16)	3.1 (0.18)	0.003*
Peer problems	2.2 (0.13)	1.6 (0.15)	0.000***	2.6 (0.17)	1.4 (0.12)	0.000***
Prosocial behaviour	7.9 (0.18)	7.3 (0.17)	0.001***	8.2 (0.16)	8.4 (0.13)	0.294

*Note.* Values in parentheses are standard errors due to clustering analysis. Australian normative SDs were converted to SE. SDQ Total difficulties range: 0 to 40. The five SDQ subscales range: 0 to 10

*BNLA* Building a New Life in Australia young refugee sample, *AUS* Australian norms

*p ≤ 0.05, ***p ≤ 0.001

Compared to Australian norms, refugee boys and girls fared comparatively well, or equivalently, on overall social and emotional functioning and subdomains, with exceptions in the 14–17 age group. Specifically, 14- to 17-year-old refugee boys reported significantly lower SDQ total total scores (i.e. higher adjustment) than Australian norms (*p* = 0.000). In contrast, 14- to 17-year-old refugee girls reported significantly higher SDQ total scores (i.e. lower adjustment) than Australian norms (*p* = 0.036).

Significant differences across the SDQ subscale domains are described below (for a detailed overview of these comparisons refer to Table 2).

##### Emotional symptoms

On the emotional symptoms subscale, refugee boys and girls did not differ significantly across ages or gender compared to Australian equivalent norms.

##### Conduct problems

On the conduct problems subscale, boys aged 11–13 and 14–17 differed significantly from their Australian age matched norms on conduct problems, wherein refugee boys reported lower levels of conduct problems (*p* = 0.000). No other significant age or gender differences emerged.

##### Hyperactivity/inattention symptoms

On the hyperactivity/inattention subscale, refugee boys across ages reported significantly lower scores than Australian age and gender equivalent norms (5-10 age group, *p* = 0.037; 11-13 age group, *p* = 0.008; 14-17 age group, *p* = 0.000). Refugee girls aged 5–10 were found to have significantly higher levels of hyperactivity and inattention compared to Australian norms (*p* = 0.023), whereas refugee girls aged 14–17 reported significantly lower levels of hyperactivity and inattention compared with Australian norms (*p* = 0.003).

##### Peer problems

Across all age groups, refugee boys and girls reported more peer difficulties than Australian age- and gender-matched norms (Boys: 5-10 age group, *p* = 0.000, 11-13 age group, *p* = 0.001, 14-17 age group, *p* = 0.000; Girls: 5-10 age group, *p* = 0.000, 11-13 age group, *p* = 0.000, 14-17 age group, *p* = 0.000).

##### Prosocial behaviours

There were no significant differences for refugee boys and girls aged 5–10 and 11–13 compared to their Australian counterparts on prosocial behaviour. However, there was a significant difference for refugee boys aged 14–17 (but not girls). This group reported higher levels of prosocial behaviour compared to Australian norms (*p* = 0.001).

#### Percentages of normal, borderline and abnormal categories on the SDQ

Table 3 shows the proportions of boys and girls categorised in the ‘Normal’, ‘Borderline’ or ‘Abnormal’ ranges on the SDQ total score and subscales. Consistently, most refugee children and adolescents reported functioning in the normal ranges (SDQ total difficulties range 75.9-93.7%). Among boys and girls of all ages, the highest rates of borderline and abnormal scores were in the peer problems domain (range 0–28.4%). For boys and girls, there was an inverse pattern for peer problems with increasing age (the under 11 age group had the highest rates of elevated scores, while the 14–17 age group had the lowest rates).

**Table 3:** Strengths and Difficulties Questionnaire (SDQ) categorisation rates for boys and girls in the Building a New Life in Australia (BNLA) young refugee sample

	Boys			Girls		
	Normal	Borderline	Abnormal	Normal	Borderline	Abnormal
Parent-Report (5–10, *n* = 216)	(*N* = 109)			(*N* = 107)		
Emotional symptoms	75.2%	7.3%	17.4%	77.3%	10.3%	12.4%
Conduct problems	76.1%	9.2%	14.7%	81.4%	9.3%	9.3%
Hyperactivity symptoms	80.7%	8.3%	11.0%	88.7%	6.2%	5.2%
Peer problems	57.8%	13.8%	28.4%	62.9%	17.5%	19.6%
Prosocial behaviour	81.7%	9.2%	9.2%	91.8%	2.1%	6.2%
SDQ total difficulties	75.9%	9.3%	14.8%	84.4%	10.4%	5.2%
Self-report (11–13, *n* = 170)	(*N* = 88)			(*N* = 82)		
Emotional symptoms	85.1%	5.7%	9.2%	75.3%	11.1%	13.6%
Conduct problems	92.0%	3.4%	4.6%	85.2%	7.4%	7.4%
Hyperactivity symptoms	93.2%	1.1%	5.7%	96.3%	1.2%	2.4%
Peer problems	83.1%	14.5%	2.4%	86.7%	13.3%	0.0%
Prosocial behaviour	94.2%	3.5%	2.3%	95.1%	1.2%	3.7%
SDQ total difficulties	90.5%	8.3%	1.2%	90.1%	3.7%	6.2%
Self-report (14–17, *n* = 243)	(*N* = 128)			(*N* = 115)		
Emotional symptoms^a^	86.7%	7.0%	6.3%	68.7%	14.8%	16.5%
Conduct problems	91.4%	4.7%	3.9%	87.0%	7.0%	6.1%
Hyperactivity symptoms	97.6%	1.6%	0.8%	95.6%	4.4%	0.0%
Peer problems	77.3%	22.7%	0.0%	72.7%	25.5%	1.8%
Prosocial behaviour	89.9%	6.2%	3.9%	91.2%	3.5%	5.3%
SDQ total difficulties^a^	93.7%	6.3%	0.0%	80.4%	15.2%	4.5%

^a^Indicates significant gender differences (*p* < 0.05) based on χ^2^ tests

In the 14–17 age group, there were significant gender differences in each of the three ‘Total Difficulties’ categories (*p* = 0.004). Inspection of the adjusted standardised residuals showed that girls were overrepresented in the ‘Borderline’ and ‘Abnormal’ categories. This suggests that more girls reported overall problems in relation to their SDQ scores compared to boys. Again, in the 14–17 age group, gender differences were evident in each of the three ‘Emotional symptoms’ categories, (*p* = 0.002). Inspection of the adjusted standardised residuals found that females were overrepresented in the ‘Abnormal’ category. This suggests more girls reported emotional problems than boys.

### Predictors of adjustment

#### Regression analyses

A series of regression models were estimated to identify risk markers for child adjustment problems as defined by SDQ total scores. These considered the range of variables across individual, family, school and community domains that were specified as exogenous predictors of SDQ scores, with each variable considered in a separate (bivariate) regression model in the first instance. In the context of either nominal or ordinal predictors with limited variability, or variables with highly skewed distributions, these were collapsed to form binary indicators and simplify the interpretation of effects. For example, the 5-point measure of caregiver-rated child health was collapsed to indicate good/fair/poor health = 1 (versus excellent/very good health = 0), while the continuous measure of years spent in refugee camps, which was characterised by limited variability among non-zero scores, was collapsed to form an indicator of 1 year or more spent in camps = 1 (versus no time or less than 1 year in camps = 0).

The results of these regression analyses are shown in Table 4. As can be seen, the largest effects were observed for parental hostility and subjective reports of child health, which were both associated with higher scores on the SDQ, suggesting greater adjustment difficulties. The bivariate models indicated smaller but significant effects for measures of academic achievement and absenteeism, as well as parental warmth. Table 4 shows results from a multiple regression model, which indicated that, while the association with parental warmth was reduced to marginal significance (*p* < 0.10) when controlling for other predictors, the aforementioned effects of parental hostility, caregiver-rated health, academic achievement and absenteeism were reduced but remained significant. In contrast, the effect of age was significant in the multiple regression but not in bivariate analyses. There was no evidence of associations with any variables in the community domain.

**Table 4:** Bivariate and multiple regressions including variables from each domain as correlates of SDQ total scores

Domain/factor	n	Bivariate regression			Multiple regression		
		Estimate	95% CI		Estimate	95% CI	
			LB	UB		LB	UB
Individual							
Age	694	−0.05	−0.12	0.03	−0.11**	−0.18	−0.05
Gender (Female)^a^	694	0.02	−0.05	0.10	0.04	−0.03	0.10
Time in refugee camp (Yes)^a^	694	−0.01	−0.10	0.09	0.00	−0.08	0.08
Caregiver ratings of child health (good/fair/poor)^a^	694	0.30***	0.22	0.39	0.22***	0.14	0.29
Number of days physical activity	694	− 0.07	− 0.16	0.03	− 0.05	− 0.12	0.03
Family							
Family structure (single parent)^a^	694	0.09†	0.00	0.18	0.05	−0.03	0.13
Parental/caregiver warmth	694	−0.12*	− 0.21	− 0.02	−0.07†	− 0.15	0.00
Parental/caregiver hostility	694	0.44***	0.34	0.53	0.39***	0.31	0.48
School							
School achievement (below average)^a^	694	0.19***	0.09	0.28	0.13**	0.05	0.21
Number of days absent	694	0.16**	0.05	0.28	0.11*	0.01	0.21
Achieved school award^b^	473	−0.02	−0.12	0.08	–	–	–
Community							
Extracurricular activities^b^	473	0.03	−0.08	0.15	–	–	–
Support from ethnic/religious community (no support)^a^	694	0.02	−0.08	0.11	0.01	−0.06	0.09
Neighbourhood feels unsafe	694	0.03	−0.05	0.11	−0.03	−0.10	0.05
People unfriendly	694	0.07	−0.03	0.18	0.03	−0.07	0.12

^a^Gender, time in refugee camp, child health, family structure, school achievement and support from ethnic/religious community were categorical variables. Their coding for the regression model is specified in brackets

^b^‘Achieved school award’ and ‘extracurricular activities’ were not included in the multiple regression model because these measures were only asked in the child self-report version (completed only by 11- to 17-year-old children), and data were thus based on a smaller sample

*CI* confidence interval, *LB* lower bound, *UB* upper bound

† *p* < 0.10; *p < 0.05; **p < 0.01; ***p < 0.001

## Discussion

We report herein, for the first time, psychosocial outcomes related to children and adolescents from the BNLA study – the largest, nationally representative Australian longitudinal study of resettled refugees and their families [1]. We explored the adjustment outcomes of children and adolescents 2–3 years following arrival to Australia. We were informed by an ecological framework that emphasised the individual (i.e. age, gender, physical health and activity, refugee camp experience), family (i.e. structure, parenting warmth and hostility), school (i.e. achievement, absenteeism), and community (i.e. extracurricular activity, neighbourhood friendliness and safety and ethnic/religious community support) domains.

The results indicated that refugee children and adolescents are adjusting soundly to their new lives in Australia, at a time when the acute stressors of resettlement are likely to have abated. The majority of children and adolescents were living in dual caregiver households and had adopted English in addition to using their caregivers’ language at home. They reported high levels of physical health and activity and engagement in extracurricular activities (e.g. dance, sports). Notably, low levels of school absenteeism were reported, along with high ratings of school achievement and many participants (up to a quarter) reported school-based awards and achievements. These outcomes may reflect the protective acculturative factors in the post-migration period documented in the literature for refugees and migrants [7, 20].

The findings from our study show that, when considered as a group, relative to non-refugee Australians, this cohort of refugee children and adolescents were generally adjusting well. In fact, 76–94% of this sample reported functioning in the normal ranges of adjustment. Using the SDQ total scores as our indicator of adjustment, we found that refugee children and adolescents did not differ from Australian age- and gender-matched norms, with exception of 14- to 17-year-old refugee boys, who reported overall higher levels of adjustment than Australian norms. One group in which refugee children and adolescents reported lower overall adjustment than Australian peers was among 14- to 17-year-old girls.

This was similarly the case across subscale measures of adjustment, including emotional difficulties, conduct problems, hyperactivity/inattention difficulties and prosocial behaviour. Refugee children and adolescents were comparable to, or had higher adjustment levels, relative to those seen in the Australian community. Among 14- to 17-year-old refugee boys in particular, a group often identified in the literature and media as at-risk for behavioural and social difficulties [16, 18], the data from this sample suggest significantly lower levels of difficulties than their Australian male counterparts. The only exception concerned 5- to 10-year-old girls, for whom caregivers reported greater levels of hyperactivity and inattention than Australian norms.

It is difficult to explicate the nuanced differences regarding gender comparisons in this study, but findings are generally consistent with prior research showing that refugee adolescent girls may be at higher risk for social and emotional difficulties than boys [20]. Although this question was not directly addressed in the current study, there are several potential reasons for such gender differences. It is possible girls may have more difficult or negative migration experiences, with a higher risk for certain traumatic events than boys (e.g. sexual violence) [45]. Further, girls may experience greater difficulties than boys in the post-migration setting, for instance, through prejudice or gender discrimination (e.g. being more identifiable as belonging to distinct cultural/religious backgrounds) [53]. Finally, adolescent girls may be more prone to internalising emotional difficulties and such risk may be particularly pronounced in refugee girls [54].

As an overall population though, refugee children and adolescents appear to be functioning soundly relative to Australian peers. This finding that young refugees function comparably despite potential adversities is suggestive of resilience among this group and might be explained in two ways. First, the positive adjustment of these young people could be related to the length of time since arriving in Australia and opportunities to acculturate within school and community contexts. This explanation is consistent with longitudinal studies that show decreases in the experience of mental health problems among resettled refugees over time [25, 55] and studies examining acculturative processes in refugees [56]. As Wave 3 represents the 2- to 3-year period following being granted a visa, it is plausible that acute social stressors, such as housing and accommodation concerns, language acquisition and school integration, are stabilised, which may have helped to promote the overall adjustment in these young people. This study did not measure the impacts of these acute stressors so careful interpretation is required, though our data support an explanation of stabilisation in the latter stages of early resettlement. The finding that the majority of refugee children and adolescents did not endorse emotional difficulties is also consistent with a multitude of adult and child studies that suggest the majority of refugees do not have psychological disorders [57].

A second possible explanation is that positive adjustment outcomes of this cohort reflect the screening and selection processes that Australia engages in, particularly in their ‘offshore’ humanitarian migration pathway. For example, the majority of young refugees in this sample had not arrived as asylum seekers, had not spent time in multiple refugee camps, and arrived accompanied. This explanation is further supported by the finding that most children in this sample were living in relatively intact dual caregiver households, which may be somewhat higher than that seen in other studies. These factors may have contributed to a better adjusted sample generally. Additionally, once granted a humanitarian visa, Australia has a well-established and often enviable Humanitarian Settlement Service programme that provides assistance to refugees with accommodation, as well as information and access to food, education, language and health services. The positive adjustment seen in these young people could be a reflection of the successes of policies and services offered as part of Australia’s settlement programme.

Notwithstanding the overall adjustment of young refugees in this sample, as a whole, they did report significantly higher levels of peer problems than comparative Australian norms. This was a consistent finding across all age groups and genders and may in part have driven the increased risk seen in the subgroup of 14- to 17-year-old refugee girls. Peer difficulties measured by the SDQ concern problems with peer interactions, forming friendships, being generally liked, picked on, and preference for being on one’s own or with adults. While difficulties with peers among young refugees was consistent, it is not clear what the conditions of such problems are, or indeed, whether these problems are in themselves a risk for, cause of adjustment difficulty or consequence of other factors (e.g. peer difficulties as an outcome of multiple school transitions or social exclusion, or an outcome of mental health problems).

Although further investigation is required to examine the nature of peer difficulties among this group of young refugees, the findings could suggest a number of relevant contextual factors. The development and maintenance of friendships, particularly in the adolescent period, is critical to social and emotional development [58]. The peer difficulties relative to Australian norms in this sample of young refugees could reflect an amplification of acculturative stress around forming and maintaining supportive peer relations (e.g. language difficulties as barriers, navigating customs around interacting with peers), as well as experiences of social exclusion, including isolation, prejudice, racism or discrimination. This is supported by studies showing that social exclusion and lack of belonging are risk factors for poor wellbeing in refugee youth [26], as well as evidence from school and community-based programmes which show that promoting social networks can enhance adjustment [59].

In addition to examining how young refugee children and adolescents compare to Australian norms, we also examined a range of individual, familial, school and community factors associated with adjustment as indicated by total SDQ scores. While existing research has identified a range of putative risk and protective factors, our study extended previous work by measuring factors systematically to assess their associations with a global assessment of functioning in young people, rather than mental health difficulties specifically. The results indicated that higher adjustment was associated with ratings of better physical health and school achievement, while poorer adjustment was associated with school absenteeism and a hostile parenting style.

Although the causal nature of these relationships cannot be established at this stage, and work is required to examine causal relationships and interactions within and across time for young refugees, these early findings suggest that certain factors can contribute to an understanding of young refugees’ adjustment during the early settlement period. Collectively, the findings suggest that risk and protective factors most proximal to the child (e.g. in what Bronfenbrenner describes as the microsystem – individual, family, school and peer networks) may play critical roles in the adjustment of young refugees in the post-migration period. From a health and settlement policy perspective, our findings suggest that individual factors, including physical health, family factors such as hostile parenting styles, and school factors such as absenteeism and achievement, may inform prevention, screening and intervention efforts (e.g. strategies to improve physical health, parenting strategies that focus on decreasing hostility and enhancing warmth and nurturance, and strategies to encourage school participation and recognise achievement). Further, our gender-specific findings regarding adjustment point to value from more focused strategies (i.e. targeting adolescent girls, and hyperactivity and inattention difficulties among younger girls, 5 to 10 years old).

### Strengths and limitations

This is the first large and broadly representative study of refugee children and adolescents resettled in Australia, which provides robust indications of adjustment among resettled young refugees. As such, the study has helped to bridge some of the disparate findings concerning mental health and well-being in prior studies. However, these findings should be considered in light of limitations. As with all cross-cultural studies, transcultural bias and translation non-equivalence associated with measures developed in western cultural settings may have affected responses. Social desirability may have also influenced reporting of positive adjustment (for example, there were only 6.1% of parents who reported overall school achievement as being ‘below or well below average’). This desire to respond with socially appropriate answers may have differentially affected refugees given the public discourse surrounding refugee resettlement in Australia. Unfortunately, investigation of culturally specific idioms of distress (e.g. somatic complaints) were beyond the scope of this study but future directions could involve mapping these findings onto culturally specific expressions of adjustment. In some instances, single item tools were the only measures available to assess some domains (e.g. physical health). Whilst this study did use reports from either caregivers or children themselves, where available or appropriate (older children as more reliable self-reporters), these are not necessarily interchangeable. Given constraints on primary data collection, information obtained directly from schools (for example, regarding school achievement) were also not available. Obtaining these may help future research validate relevant findings. Finally, our statistical analyses incorporated adjustments for clustering within families, but were unable to model clustering within schools or neighbourhoods. While multilevel frameworks provide enhanced correspondence with ecological models, the current approach was ‘single-level’ and may be characterised by underestimates of standard errors (e.g. due to non-independence of families within neighbourhoods). The bivariate regression analyses were accompanied by a multiple regression model that included predictor variables simultaneously. This was intended to minimise the risk of confounded associations, but should be viewed cautiously given limitations in hypotheses regarding causal structures. Until more accurate predictive models can be determined, it is important to recognise these individual factors as conferring risk or protection rather than being a necessary condition for good or poor adjustment [60–62].

## Conclusions

This was the first study to report outcomes for children and adolescents from the longitudinal BNLA study. Generally, refugee children and adolescents in the study reported adjusting soundly in the 2 to 3-year period after arrival in Australia. While it cannot be ignored that refugee children and adolescents do experience vulnerability on account of pre- and post-migration adversities, we present preliminary evidence suggesting that parental capability, physical health, school participation and achievement may be linked to improved settlement outcomes for this population. Further research may seek to replicate these findings and examine the nature of peer difficulties to inform how these are linked to adjustment outcomes. Contextual factors, including the settlement policies of host countries and the host country itself, should also be considered when assessing post-settlement adjustment outcomes in young refugees.
